# The Hungry Heart: Managing Cardiogenic Shock in Patients with Severe Anorexia Nervosa—A Case Report Series

**DOI:** 10.3390/jcm14114011

**Published:** 2025-06-05

**Authors:** Manuela Thienel, Rainer Kaiser, Jonas Gmeiner, Martin Orban, Stefan Kääb, Tobias Petzold, Steffen Massberg, Clemens Scherer

**Affiliations:** 1 Department of Cardiology, Ludwig-Maximilians-University Hospital, Ludwig Maximilians University, 81377 Munich, Germanyclemens.scherer@med.uni-muenchen.de (C.S.); 2 DZHK (German Centre for Cardiovascular Research), Partner Site Munich Heart Alliance, 80336 Munich, Germany; 3 Kardiologie Ammer-Lech, 86911 Dießen am Ammersee, Germany; 4 Department of Cardiology, Angiology and Intensive Care Medicine, Deutsches Herzzentrum der Charité, University Hospital Berlin, Campus Benjamin Franklin, 12203 Berlin, Germany; 5 DZHK (German Centre for Cardiovascular Research), Partner Site Berlin, 10785 Berlin, Germany; 6 Friede Springer, Centre of Cardiovascular Prevention at Charité, Charité University Medicine Berlin, 12203 Berlin, Germany

**Keywords:** anorexia nervosa, cardiogenic shock, malnutrition, heart failure, mechanical circulatory support, case report

## Abstract

**Background:** Cardiogenic shock is a life-threatening condition characterized by the failure of the heart to maintain adequate circulation, leading to multi-organ dysfunction. While it is most commonly associated with acute myocardial infarction or cardiomyopathies, cardiogenic shock can also arise in unusual settings, such as severe malnutrition in patients with anorexia nervosa, a psychiatric disorder characterized by extreme restriction of food intake. **Methods:** Here, we describe the management of three patients with anorexia nervosa and severe cardiogenic shock, who were treated in our cardiological intensive care unit between December 2022 and January 2025. Two patients were successfully resuscitated after experiencing cardiac arrest, and two required mechanical circulatory support, including Venoarterial Extracorporeal Membrane Oxygenation and microaxial flow pump. The patients presented with a range of complications including multi-organ failure and respiratory distress. Due to the fragile balance between intensive cardiac and nutritional management, as well as the comorbidity of chronic malnutrition, therapeutic decisions were made carefully, including cautious electrolyte management, targeted nutritional therapy, and the use of advanced circulatory support. **Conclusions**: The treatment approach and beneficious outcomes underline the necessity of a multidisciplinary strategy in managing these critically ill patients with complex, interwoven pathologies. Our experience suggests that early recognition of cardiogenic shock and timely intervention with mechanical circulatory support may significantly improve patient survival in this high-risk cohort. Careful management of nutritional therapy and supplementation of trace elements and vitamins is crucial.

## 1. Introduction

Severe malnutrition, particularly in the context of anorexia nervosa (AN), can lead to significant metabolic and cardiovascular disturbances. One of the most dangerous complications of malnutrition is the development of cardiogenic shock (CS), a condition in which the heart is unable to provide sufficient cardiac output, leading to systemic hypoperfusion. Despite its rarity, CS in patients with severe AN is a critical condition that can be fatal if not promptly recognized and treated.

The pathophysiology behind this association is multifactorial, including myocardial dysfunction, electrolyte imbalances, and the effects of extreme weight loss on cardiac structure and function [1,2]. Here, we present three patients who developed CS due to severe AN, offering a valuable insight into this rare yet potentially fatal complication and its therapeutic management.

## 2. Cases

### 2.1. Case 1

A 54-year-old female patient with a history of restricting-type AN (current BMI 12 kg/m^2^) was transferred to our department due to progressive CS of unclear etiology (Table 1). 

When asked, the patient’s relatives denied vomiting or laxative/diuretic abuse. She was admitted after a fall at home, resulting in a multi-fragmentary left femoral fracture. During emergency surgery, hypokalemia, hyponatremia, and hypocalcemia led to asystole, which progressed to ventricular fibrillation. Immediate cardiopulmonary resuscitation was performed, and after 5 min, return of spontaneous circulation was achieved. Coronary angiography excluded coronary artery disease. Echocardiography revealed severely impaired left ventricular ejection fraction ((LVEF) of 15%) with akinesia of the left ventricular septum and free walls. There was no indication of classic dilated cardiomyopathy, valvular heart disease, or stress-induced (Takotsubo) cardiomyopathy. Likewise, no signs of primary arrhythmia or QT interval prolongation were detected. The patient was in progressive CS, requiring high doses of catecholamines (norepinephrine 2 mg/h, epinephrine 1.4 mg/h, dobutamine 20 mg/h). Upon arrival to our facility, the patient was intubated and mechanically ventilated.

Laboratory findings indicated multi-organ failure with severe lactic acidosis (6.5 mmol/L), oliguria, spontaneous INR under liver failure, and thrombocytopenia. Additional abnormalities included hypokalemia, hypocalcemia, hypophosphatemia, and hypoproteinemia. Bilateral pleural effusions and atelectasis were confirmed by ultrasound, and pleural puncture was performed. Continuous venovenous hemodialysis (CVVHD) was initiated for oliguria. Antibiotics (Piperacillin/Tazobactam) were maintained due to suspected pneumonia and septic shock.

High-dose supplementation of trace elements and vitamins was initiated along with a calorie-adjusted diet. To prevent refeeding syndrome, nutrition started at 10 kcal/kg body weight, gradually increasing to an amount adjusted to the patient’s ideal weight. Phosphate supplementation was provided. Over time, left ventricular function progressively improved, leading to complete normalization, allowing for the gradual reduction in catecholamine support.

Following the normalization of leukocytes, platelet count rose significantly after 2 days. On the same day, a modular hip prosthesis was implanted. Postoperatively, additional transfusions were required due to hemoglobin drop and catecholamine dependency.

After sedation reduction, the patient showed a satisfactory awakening response and was successfully extubated. However, due to continued protein deficiency and hypotensive episodes, additional plasma transfusions were given. As the patient showed increasing respiratory exhaustion and muscle weakness, re-intubation was performed 2 days later. Since the patient was almost continuously intubated during her stay in our clinic, a sufficient psychiatric evaluation could not be carried out. However, this was strongly recommended for further treatment as soon as the patient regained consciousness. Two days after re-intubation, the patient was then transferred back to the receiving hospital for the weaning process. At the last follow-up, 26 months after discharge, the patient was alive and reported NYHA Class II.

### 2.2. Case 2

A 25-year old male patient with severe AN was transferred to our intensive care unit, following a life-threatening cardiac arrest event (Table 1). The patient was found collapsed at home, and resuscitation was initiated by the emergency medical team after finding the patient unresponsive with irregular breathing. Upon arrival, the patient was in a state of profound hypoglycemia (serum glucose 38 mg/dL). Cardiopulmonary resuscitation was performed for approximately 70 min, with a cumulative dose of 11 mg of adrenaline administered, ultimately resulting in the return of spontaneous circulation with insufficient blood pressure.

Echocardiography revealed cardiac pump failure with severely impaired left ventricular ejection fraction with only minimal myocardial contraction at the apex. There was no evidence of dilated cardiomyopathy, valvular heart disease, or Takotsubo cardiomyopathy. Likewise, no signs of primary arrhythmia or QT interval prolongation were detected. Venoarterial extracorporeal membrane oxygenation (VA-ECMO) was initiated in our emergency department. Coronary angiography ruled out coronary artery disease and computed tomography angiography excluded pulmonary embolism but revealed active bleeding from the right external iliac artery, as well as a hematoma in the left inguinal region and a diffuse hemorrhage. The patient was transferred to our cardiac intensive care unit, intubated, and dependent on catecholamines, with low-flow support from VA-ECMO. Clinically, the patient presented in a pronounced cachectic state (BMI 15.6 kg/m^2^). According to the patient’s aunt, the patient has been suffering from a severe eating disorder for several years, with multiple outpatient and inpatient treatments. In recent weeks, the patient has been almost exclusively consuming low-calorie vegetable juices and smoothies. Self-induced vomiting or laxative and diuretic abuse was not known. Laboratory tests indicated the onset of liver failure with coagulopathy with significantly prolonged INR and PTT values and pancytopenia with severe anemia. Fibrinogen, prothrombin complex concentrates (PPSBs), platelet, and erythrocyte concentrates were transfused, leading to stabilization of coagulation.

Initially, a cautious parenteral nutrition regimen was started to prevent refeeding syndrome, which was gradually increased and supplemented with enteral nutrition after a few days. A jejunal feeding tube was placed after approximately 2 weeks due to reflux.

Over the following days, there was a marked decrease in diuresis, necessitating CVVHD. With careful negative fluid balance, both pulmonary edema and peripheral edema showed gradual regression. The bone marrow failure, likely secondary to malnutrition, along with superficial bleeding from VA-ECMO cannulation sites, hemolysis, and platelet consumption due to VA-ECMO support, led to a significant transfusion requirement for platelets and erythrocyte concentrates. Simultaneously, repeated coagulation optimization was necessary, including the administration of Factor XIII and fibrinogen.

In the following weeks, the patient exhibited a gradual improvement in cardiac function with increased blood pressure amplitude, allowing for VA-ECMO removal after about 2 weeks. The patient’s condition improved with progressive recovery of cardiac output and cessation of catecholamine support. Psychiatric evaluation was significantly limited due to general weakness and tracheostomy. Nevertheless, the patient declined psychotropic medication by shaking his head. Different pharmacological treatment options were discussed; however, the patient and his aunt expressed a preference for initiating psychological therapy in advance. Despite ongoing challenges, including respiratory and infective complications with Enterococcus faecium and later Candida glabrata as well as impaired liver function, the patient gradually stabilized. A few weeks later, the patient was able to undergo intermittent high-flow nasal cannula oxygen therapy and was de-cannulated in the end. The patient was eventually transferred for neurological rehabilitation after a prolonged period of intensive care. At this point, the cardiac pump function had almost fully recovered and was only minimally impaired. Within the next months, the patient developed severe secondary sclerosing cholangitis, but was alive at the last follow-up, 22 months after discharge.

### 2.3. Case 3

A 55-year old male patient was transferred to our facility in critical condition, presenting with mixed septic and cardiogenic shock (SCAI Stage E) and a history of severe restricting-type AN (current BMI approximately 12.5 kg/m^2^, Table 1). Self-induced vomiting or laxative and diuretic abuse was not known. Initial symptoms included dizziness, deterioration of general condition, and coughing with brownish sputum, in the context of an infection and hypoglycemia. Dilated cardiomyopathy had already been described three years ago, initially presenting with severely reduced pump function of approximately 25%, which improved to 50% with heart failure therapy. At the time of admission to the referring hospital, left ventricular ejection fraction (LVEF) was severely reduced, estimated at approximately 5%. There was no evidence to suggest valvular pathology, or stress-induced (Takotsubo) cardiomyopathy. Similarly, no indications of a primary arrhythmia or QT interval prolongation were observed. Due to increasing catecholamine requirements, with norepinephrine doses escalating to 8 mg/h, the patient underwent implantation of a VA-ECMO.

Upon arrival at our intensive care unit, the patient was alert, oriented, and breathing spontaneously with an oxygen mask delivering approximately 8 L/min. Initial norepinephrine infusion was around 4 mg/h. After volume substitution, catecholamine doses were significantly reduced. Respiratory function improved under Continuous Positive Airway Pressure (CPAP) therapy, and the patient’s condition remained stable, avoiding the need for endotracheal intubation.

On the following day, the patient underwent uncomplicated bilateral pleural drainage due to large pleural effusions, following transfusion of platelet concentrates. In light of a history of invasive aspergillosis and infection, along with brownish sputum, treatment was initiated with Piperacillin/Tazobactam and Voriconazole. Infection markers decreased under this therapy. The nutrition team recommended gradual increases in calorie intake and vitamin supplementation, while iron supplementation was also initiated.

Further evaluation of initial pancytopenia was performed with a bone marrow biopsy, which did not show evidence of hematologic malignancy. Due to recurring thrombocytopenia, Romiplostim was administered. As renal function worsened and progressive volume overload was noted, CVVHD was initiated.

The patient’s psychiatric assessment was limited due to being in severe cardiogenic shock and receiving ECMO support. In his medical history, he had previously been treated with SSRIs and mirtazapine for depression. Until now, he had declined recommended inpatient psychiatric treatment; however, he has now expressed willingness to undergo psychiatric care after the acute event.

Due to persistently severely reduced left ventricular function, it was decided to implant an Impella 5.5 device via an axillary access route. The patient stabilized progressively, allowing for decannulation of the VA-ECMO on the 7th day after implantation. With increasing caloric intake and vitamin supplementation, there was a marked improvement in pump function with an increase in left ventricular wall thickness. After continued weaning from circulatory support, the Impella device was explanted on day 15 after implantation. After the sedation was discontinued, the patient exhibited a delayed awakening response as well as severe critical illness polyneuropathy. During the course, the patient was transferred to neurological early rehabilitation. On the day of transfer, the cardiac pump function had recovered to 50%. Nonetheless, the patient passed away 2 months later at an external facility.

## 3. Therapy Recommendation

AN is a rare etiology of cardiogenic shock and should therefore be regarded as a diagnosis of exclusion. Other more prevalent causes—such as acute or chronic coronary syndrome, acutely decompensated heart failure, fulminant myocarditis, advanced valvular disease, and significant arrhythmias—should be systematically excluded. Evidence about the optimal management of AN in the intensive care unit is scarce. So far, only one study was conducted in France, which analyzed the prevalence and outcome of patients with AN in the intensive care unit. Altogether, the prevalence of AN was very low with only 68 patients in 30 intensive care units and a mortality rate of 10%. Cardiac failure was reported in three patients and cardiac arrest in one patient [3].

For patients with AN and CS, we followed the same therapy principles as for other patients with CS [4]: As first-line catecholamines, we utilized dobutamine and norepinephrine. For progressive cardiogenic shock, mechanical circulatory support was discussed on an individual basis bearing in mind possible vascular complications. Restoring cellular energy metabolism may improve heart failure in ways that differ from those observed in other forms of cardiogenic shock, which should be kept in mind when evaluating mechanical support devices. Furthermore, patients with AN and CS are at increased risk of arrhythmias due to electrolyte disturbances; therefore, special care—exceeding standard heart failure guideline recommendations—should be taken as outlined in the following paragraphs.

Refeeding syndrome is a life-threating disease in patients with AN and should be avoided by using proper nutrition guidelines. Therefore, regular checks of electrolytes, substitution of phosphorus, and cautious refeeding are of utmost importance. If refeeding syndrome should develop, treatment includes reduction in carbohydrate intake, a correction of hypophosphatemia, and a parenteral substitution of vitamins and trace elements [5].

Altogether, we used the following guidelines for treating patients with AN and cardiogenic shock, which are based on the recommendations of the American Society for Parenteral and Enteral Nutrition as well as the guidelines of the German Society for Nutritional Medicine [6,7]:

- Continuous hemodynamic and ECG monitoring.
- Central venous access.
- Daily 12-lead ECG.
- Daily transthoracic echocardiography.
- Regular checks of glucose, electrolytes (potassium, sodium, phosphorus, magnesium, and calcium), kidney tests (blood urea nitrogen, creatinine), liver tests (AST, ALT, bilirubin), thyroid function tests (TSH, fT3, fT4) complete blood count, albumin, total proteins, C-reactive protein, 25-hydroxyvitamin D3 levels.
- Regular checks of hydration status.
- Mean arterial pressure aim of ≥ 65 mmHg.
- First line catecholamines: dobutamine and norepinephrine.
- Mechanical circulatory support for patients with progressive cardiogenic shock.
- Initial phase with high catecholamine support: parenteral nutrition, in case of reduced catecholamine support enteral nutrition.
- Calorie intake:
  ○ Day 1–3: 5–10 kcal/kg BW/d.
  ○ Day 4–6: 10–20 kcal/kg BW/d.
  ○ Day 7–9: 20–30 kcal/kg BW/d.
  ○ Day 10 and more: Full recommended calorie intake.
- Fluid replacement:
  ○ Day 1–3: 20–25 mL/kg BW/d.
  ○ Day 4–6: 25–30 mL/kg BW/d.
  ○ Day 7 and more: 30–35 mL/kg BW/d.
- Thiamine substitution (200–300 mg) on days 1–5.
- Phosphorus substitution: 750–1500 mg daily.
- Daily substitution of polyvitamine and multi-trace element solution.
- Electrolyte substitution if necessary.
- Psychotherapeutic interventions if possible.

## 4. Discussion

Severe AN is associated with an approximately fivefold increased mortality risk, driven both by significantly elevated suicide rates and severe cardiovascular complications [8]. All three of our patients with severe AN and CS demonstrated full or near-complete recovery of cardiac function following comprehensive intensive care, including cautious nutritional rehabilitation and, when necessary, mechanical circulatory support. Two patients were alive two years after discharge, while one patient unfortunately passed away 2 months later at an external facility. A review of the literature shows that although cardiomyopathy and CS are rare but severe complications of AN, favorable outcomes can be achieved with standard heart failure therapy [9], even in cases requiring extracorporeal support [10]. However, certain cases highlight the importance of tailored refeeding strategies, such as one report describing fat emulsion-induced midventricular obstruction caused by cardiac hypercontraction, myocardial thickening, and volume depletion [11]. These findings underline the necessity of a multidisciplinary approach with close cardiac monitoring and individualized nutritional management in critically ill AN patients.

**Figure 1:**
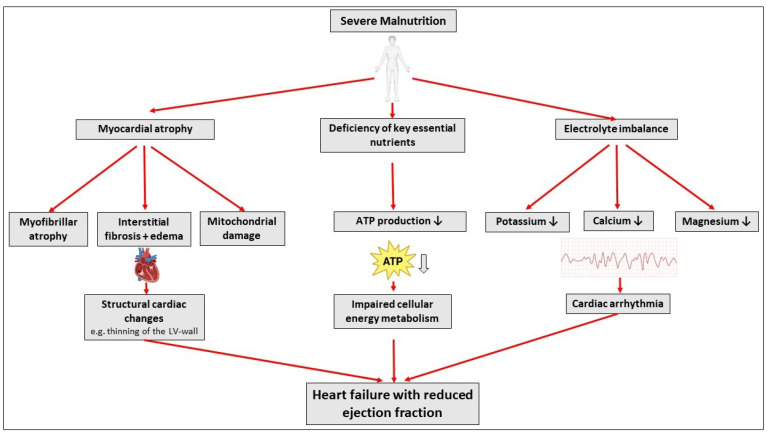
Pathophysiological mechanisms contributing to cardiomyopathy in anorexia and malnutrition (created with Biorender).

The underlying mechanisms that contribute to the development of heart failure in these patients are multifactorial (Figure 1). Malnutrition can directly affect myocardial function by inducing a state of myocardial atrophy. The heart undergoes structural changes, with the left ventricle often exhibiting thinning of the myocardial walls, which is accompanied by a reduction in myocardial contractility [1]. In the cases presented, all three patients exhibited severely reduced left ventricular function, with an ejection fraction of less than 15%. Structural myocardial changes in AN resemble those seen in cardiomyopathies, including mitochondrial damage, fibrosis, myofibrillar atrophy, and interstitial edema [2,12]. The pathophysiology of myocardial dysfunction in AN patients is related to nutritional deficiency, particularly the lack of essential fatty acids, proteins, and vitamins such as thiamine and B vitamins, which are crucial for cellular energy metabolism in cardiomyocytes [13,14]. In addition, phosphate plays a crucial role in the pathophysiology of cardiac complications in AN as it is essential for the production of adenosine triphosphate (ATP), the primary energy carrier in cells. During nutritional rehabilitation, the sudden intake of carbohydrates triggers insulin release, which shifts phosphate, potassium, and magnesium into cells for ATP synthesis. This can lead to severe hypophosphatemia, a hallmark of refeeding syndrome, potentially leading to arrhythmias and even sudden cardiac death [15]. In addition to structural cardiac changes in AN, electrolyte imbalances, including hypokalemia, hypocalcemia, and hypomagnesemia, frequently occur in AN, particularly in the binge–purge type. Recurrent episodes of self-induced vomiting, laxative, and/or diuretic abuse, which are characteristic of the binge–purge subtype, can lead to significant potassium, calcium, and magnesium losses. These electrolyte disturbances can severely impair cardiac electrophysiology by increasing myocardial irritability and impairing electrical conduction [16,17]. Hypokalemia, in particular, is closely associated with QT prolongation, involving the risk of torsades de pointes and sudden cardiac death. Various other forms of arrhythmias, including bradyarrhythmias, ventricular tachycardia, and conduction disturbances, can be exacerbated by persistent purging behavior [18,19]. Although all three patients described here were diagnosed with the restrictive subtype of AN and both patients and their families denied any binge–purge behaviors, the first patient, in particular, presented with severe electrolyte disturbances that initially resulted in asystole, followed by ventricular tachycardia. The possibility that undetected or undisclosed binge–purging behavior may have been involved cannot be ruled out.

Although AN is commonly perceived as a predominantly female disorder, two of the three patients described in this case series were male. Emerging evidence indicates that male patients are often diagnosed at a more advanced stage of the illness, frequently presenting with more severe complications [20]. This diagnostic delay may be partly attributed to the fact that the typical clinical features of AN are traditionally framed around female presentations. In addition, information material is largely designed with a female audience in mind, potentially contributing to the under-recognition of the disorder in males. Consequently, healthcare providers may be less likely to suspect AN in male patients. Although recognition and diagnosis in men often occur later, current data suggest that mortality rates are similarly high in male and female patients—though this conclusion remains tentative due to limited data [21,22]. In the cases presented here, all patients were diagnosed several years prior, making it difficult to assess whether male sex contributed to a delayed diagnosis in these specific instances.

In cases of CS, MCS devices such as VA-ECMO and microaxial flow pump are often used as life-saving interventions [4,23,24]. However, the application of these technologies in severely malnourished patients presents several challenges. A major concern is the vascular access for these devices. Patients with AN frequently have small and fragile vasculature, making the insertion of VA-ECMO cannulas or microaxial flow pump devices difficult. In our case series, the decision to use VA-ECMO in the second and third cases was complicated by the patient’s small vessel size, which necessitated careful selection of cannulation sites and continuous monitoring for complications such as bleeding or thrombosis. Moreover, coagulopathy is a common complication in AN patients, especially those with bone marrow failure secondary to malnutrition. Additionally, the consumption of platelets and erythrocytes due to prolonged VA-ECMO support further complicates the patient’s care. In these cases, frequent blood product transfusions and coagulation optimization are often required to maintain hemostasis and avoid bleeding, underlining the need for careful management of hemostatic disturbances in AN patients undergoing MCS.

## 5. Conclusions

CS in AN presents significant challenges in diagnosis and management. The mechanisms of cardiac dysfunction are complex, involving myocardial atrophy, electrolyte imbalances, and the effects of starvation on myocardial structure. The use of MCS, such as VA-ECMO and microaxial flow pump, is a valuable tool to support these critically ill patients, but it is complicated by factors such as small vessel size and coagulopathy. Additionally, the risk of refeeding syndrome necessitates careful nutritional management to avoid severe complications. Despite the limitations of a small sample size and the lack of long-term follow-up on LV function or comparative data, this article demonstrates that CS in severe AN can be successfully treated and offers a practical framework for managing these patients, particularly in intensive care settings. This could be influential in clinical environments, guiding practitioners on how to approach similar cases, especially in terms of nutritional management and the use of advanced life support technologies. However, a multidisciplinary approach involving cardiologists, intensivists, nutritionists, and infectious disease specialists is essential. Given the complexities of this condition, further research into the mechanisms underlying cardiac dysfunction in AN, as well as the safest methods for MCS and nutritional management, is essential.

## Figures and Tables

**Table 1:** Clinical characteristics and findings of study participants.

	Patient 1	Patient 2	Patient 3
Age (years)	54	25	55
Gender	female	male	male
Pre-existing conditions	stroke	schizophrenia	dilated cardiomyopathy, invasive aspergillosis
Type of anorexia (restricting/binge eating)	restricting	restricting	restricting
Medication on admission	-	-	acetylsalicylic acid, mirtazapine
BMI on admission (kg/m^2^)	12.0	15.6	12.5
SOFA II score on admission	20	16	13
SCAI stage admission	D	D	E
SCAI stage max ICU stay	D	D	E
Cardiopulmonary resuscitation (OHCA/IHCA)	IHCA	OHCA, IHCA	-
Duration of ICU stay (days)	12	58	32
Duration of hospital stay (days)	12	58	32
Systolic blood pressure on admission (mmHg)	97	82	83
Diastolic blood pressure on admission (mmHg)	72	52	73
Heart rate on admission (bpm)	89	79	109
LVEF on admission (%)	15	20	5
LVEF on ICU discharge (%)	60	45	60
Mechanical ventilation (duration in days)	13	18	12
Laboratory values on admission			
Hemoglobin (g/dL)	9.4	4.8	7.8
pH	7.31	7.65	7.39
Lactate (mmol/L)	5.3	2.5	3.4
Potassium (mmol/L)	4.5	3.9	5
Sodium (mmol/L)	136	132	140
Phosphorus (mg/dL)	0.94	3.6	5.4
Magnesium (mmol/L)	4.7	3.28	0.96
Calcium (mmol/L)	2.21	1.65	1.89
Blood urea nitrogen (mg/dL)	11	4.4	3.1
Creatinine (mg/dL)	0.9	0.6	0.8
AST (U/L)	85	918	1208
ALT (U/L)	39	681	309
Bilirubin (md/dL)	1.1	0.5	0.6
TSH (µg/mL)	0.65	2.04	0.75
fT3 (pg/mL)	1.1	1.2	1.1
fT4 (ng/dl)	0.9	0.5	1.4
Albumin (g/dL)	2.5	2.3	2.2
Total protein (g/dL)	3.8	2.8	3.3
CRP (mg/dL)	14.5	<0.1	6.2
Vitamin D (ng/mL)	<3	<3	11.3
VA-ECMO duration	-	10 days	8 days
Impella (duration)	-	-	16 days
Renal replacement therapy (duration)	5 days	40 days	2 days
Laboratory values day 1–7			
Lactate max on day 1 (mmol/L)	6.5	4.1	3.4
Lactate max on day 2 (mmol/L)	4.3	4.5	3.3
Lactate max on day 3 (mmol/L)	3.2	3.2	1.3
Lactate max on day 4 (mmol/L)	2.3	1.8	1.4
Lactate max on day 5 (mmol/L)	2	1.6	1.8
Lactate max on day 6 (mmol/L)	1.7	1.7	1.3
Lactate max on day 7 (mmol/L)	1.6	1.3	1.9
Mean Potassium day 1 (mmol/L)	4.1	3.5	4.4
Mean Potassium day 2 (mmol/L)	3.7	4.3	4.6
Mean Potassium day 3 (mmol/L)	3.8	4	4.2
Mean Potassium day 4 (mmol/L)	3.7	4.5	4.3
Mean Potassium day 5 (mmol/L)	3.9	4.2	4.1
Mean Potassium day 6 (mmol/L)	4.6	4.3	4
Mean Potassium day 7 (mmol/L)	4.2	4.4	4.5
Mean phosphorus day 1 (mg/dL)	4.7	3.3	4.95
Mean phosphorus day 2 (mg/dL)	2.85	3.8	5.35
Mean phosphorus day 3 (mg/dL)	1.95	4	5.1
Mean phosphorus day 4 (mg/dL)	1.6	3.4	4.9
Mean phosphorus day 5 (mg/dL)	2.35	2.3	4.6
Mean phosphorus day 6 (mg/dL)	2.2	4.1	2.6
Mean phosphorus day 7 (mg/dL)	3.6	5	1.7
Mean albumin day 1 (g/dL)	2.5	2.65	2.45
Mean albumin day 2 (g/dL)	3.1	n.a.	2.6
Mean albumin day 3 (g/dL)	2.7	n.a.	2.6
Mean albumin day 4 (g/dL)	3.4	3.2	2.3
Mean albumin day 5 (g/dL)	3.2	3.3	2.3
Mean albumin day 6 (g/dL)	n.a.	3.6	2.3
Mean albumin day 7 (g/dL)	3.2	3.2	n.a.
AST day 1 (U/L)	85	918	1208
AST day 2 (U/L)	74	825	504
AST day 3 (U/L)	56	333	423
AST day 4 (U/L)	290	164	340
AST day 5 (U/L)	121	82	259
AST day 6 (U/L)	81	52	159
AST day 7 (U/L)	86	32	87
ALT day 1 (U/L)	39	681	309
ALT day 2 (U/L)	32	570	195
ALT day 3 (U/L)	32	358	183
ALT day 4 (U/L)	90	282	157
ALT day 5 (U/L)	73	207	133
ALT day 6 (U/L)	61	147	97
ALT day 7 (U/L)	61	100	24
Creatinine day 1 (mg/dL)	0.9	0.6	0.9
Creatinine day 2 (mg/dL)	0.8	0.8	1.1
Creatinine day 3 (mg/dL)	0.6	1.2	1.1
Creatinine day 4 (mg/dL)	0.7	1.3	1.1
Creatinine day 5 (mg/dL)	0.7	1.2	0.9
Creatinine day 6 (mg/dL)	0.7	1.4	0.6
Creatinine day 7 (mg/dL)	0.7	1.9	0.6
Vasopressors (median dose in mg/h or µg/h)			
Norepinephrin day 1	1.5	0.4	1.1
Norepinephrin day 2	1.2	0.4	1
Norepinephrin day 3	1.1	0.5	1
Norepinephrin day 4	0.9	0.5	0.8
Norepinephrin day 5	0.3	0.2	1
Norepinephrin day 6	0.2	0.002	0.6
Norepinephrin day 7	-	0.002	0.2
Epinephrin day 1	0.7	0.1	-
Epinephrin day 2	-	-	-
Epinephrin day 3	-	-	-
Epinephrin day 4	-	-	-
Epinephrin day 5	-	-	-
Epinephrin day 6	-	-	-
Epinephrin day 7	-	-	-
Dobutamin day 1	25	-	25
Dobutamin day 2	30	-	20
Dobutamin day 3	30	-	20
Dobutamin day 4	20	10	20
Dobutamin day 5	15	10	20
Dobutamin day 6	10	10	15
Dobutamin day 7	-	10	-
Vasopressin day 1	-	-	0.8
Vasopressin day 2	-	-	-
Vasopressin day 3	-	-	-
Vasopressin day 4	-	-	-
Vasopressin day 5	-	-	-
Vasopressin day 6	-	-	-
Vasopressin day 7	-	-	-
Calorie intake (kcal)			
Calorie intake day 1	-	-	1029
Calorie intake day 2	135	1365	2016
Calorie intake day 3	277.5	2415	1806
Calorie intake day 4	2041.2	3120.09	2268
Calorie intake day 5	2922.75	3580.74	2793
Calorie intake day 6	2553.2	3537.165	2367.75
Calorie intake day 7	2046	3591.255	3024
Fluid intake (mL)			
Fluid intake day 1	4808.72	8922.86	6215.14
Fluid intake day 2	3233.43	5483.11	3118.8
Fluid intake day 3	3969.16	2607.11	4553.32
Fluid intake day 4	2591.91	2679.89	5310.33
Fluid intake day 5	3101.56	4097.92	7415.82
Fluid intake day 6	2499.09	3111.41	5010.53
Fluid intake day 7	2607.26	3810.69	5270.51
Potassium intake (mval)			
Potassium intake day 1	24.8	157.5	25.5
Potassium intake day 2	81	187.3	34
Potassium intake day 3	144	59	74.4
Potassium intake day 4	106.2	72	49.5
Potassium intake day 5	157	48	233
Potassium intake day 6	124	95.7	284
Potassium intake day 7	72	40.5	294
Phosphorus intake (mmol)			
Phosphorus intake day 1	0	0	0
Phosphorus intake day 2	20	40	0
Phosphorus intake day 3	48	24	0
Phosphorus intake day 4	0	24	0
Phosphorus intake day 5	96	24	0
Phosphorus intake day 6	24	72	0
Phosphorus intake day 7	48	48	36
Polyvitamine and multi-trace element solution day 1–7	yes	yes	yes
Vitamin B1 intake (mg)			
Vitamin B1 intake day 1	100	200	400
Vitamin B1 intake day 2	800	300	400
Vitamin B1 intake day 3	2000	400	400
Vitamin B1 intake day 4	1500	400	400
Vitamin B1 intake day 5	1500	400	0
Vitamin B1 intake day 6	1000	400	0
Vitamin B1 intake day 7	0	400	0
Vitamin D intake (IE)			
Vitamin D intake day 1	0	0	20000
Vitamin D intake day 2	0	0	1000
Vitamin D intake day 3	0	0	1000
Vitamin D intake day 4	0	1000	1000
Vitamin D intake day 5	0	1000	1000
Vitamin D intake day 6	0	1000	1000
Vitamin D intake day 7	0	1000	1000
CPC on discharge	1	1	3
Final diagnosis	cardiogenic shock due to severe malnutrition in the context of AN	OHCA with cardiogenic shock due to severe malnutrition in context of AN	cardiogenic shock due to severe malnutrition in context of AN
Last follow-up visit	alive	alive	dead

## Data Availability

The original data presented in this study are included in the article. Further inquiries can be directed to the corresponding author.

## Abbreviations

The following abbreviations are used in this manuscript:

AN	Anorexia nervosa
BMI	Body mass index
CPAP	Continuous positive airway pressure
CS	Cardiogenic shock
CVVHD	Continuous venovenous hemodialysis
LV-EF	Left ventricular ejection fraction
MCS	Mechanical circulatory support
VA-ECMO	Venoarterial extracorporeal membrane oxygenation
